# Supplementary material for: Systematic development and feasibility testing of a multibehavioural digital prehabilitation intervention for patients approaching major surgery (iPREPWELL): A study protocol
Source: PLoS One. 2022 Dec 27;17(12):e0277143. doi: 10.1371/journal.pone.0277143 (PMC9794053; doi:10.1371/journal.pone.0277143)
Supplement: S1 File — (DOCX) [file pone.0277143.s001.docx]

## COM-B Self-Evaluation Questionnaire

When it comes to you personally changing various lifestyle behaviours (e.g., increasing physical activity levels, stopping smoking) before your operation, what do you believe you need to be able to do it?

Please circle any of the items on the list that you think apply to you. You can circle as many or as few as you think appropriate. Some of the items may look strange, but that is just because we need to cover all areas – some which may not apply to you.

For each item you circle, could you also say why you think it might be important for you in the free text box provided beneath.

I would have to....

Capability

| 1. | Know more about why it is important | e.g. have a better understanding of the benefits of making lifestyle changes before my operation |
| --- | --- | --- |
|  |  | |
| 2. | Know more about how to do it | e.g. have a better understanding of how to effectively make lifestyle changes prior to my operation |
|  |  | |
| 3. | Have better physical skills | e.g. acquire/develop new skills to make lifestyle changes before my operation and overcome challenges associated with that |
|  |  | |
| 4. | Have better mental skills | e.g. learn how to reduce the likelihood of relapsing when attempting to make and maintain lifestyle changes before my operation |
|  |  | |
| 5. | Overcome physical limitations | e.g. continue to make changes to my lifestyle when feeling tired and stressed |
|  |  | |
| 6. | Overcome mental obstacles | e.g. overcome the urge to give up on making lifestyle changes before my operation when faced with challenges |
|  |  | |
| 7. | Have more physical stamina | e.g. develop greater capacity to maintain physical effort, particularly when faced with a personal challenge or obstacle |
|  |  | |
| 8. | Have more mental stamina | e.g. develop greater mental capacity to make lifestyle changes before my operation even when lacking in motivation and faced with challenges |
|  |  | |

I would have to....

Opportunity

| 9. | Have more time to do it | e.g. dedicate more time to making lifestyle changes before my operation |
| --- | --- | --- |
|  |  | |
| 10. | Have the necessary materials/resources | e.g. have the equipment or resources to help me make lifestyle changes before my operation |
|  |  | |
| 11. | Have patients around me doing the same thing | e.g. be part of a group of patients who are also trying to make changes to their lifestyle before their operation |
|  |  | |
| 12. | Have triggers to prompt me | e.g. have reminders at specific times to prompt me to make the lifestyle changes I would like to, to reach my goals |
|  |  | |
| 13. | Have support from others | e.g. have healthcare professionals, other patients or family members supporting me to make lifestyle changes before my operation |
|  |  | |

I would have to....

Motivation

| 14. | Feel that I want to do it enough | e.g. feel a sense of pleasure or satisfaction from making lifestyle changes before my operation |
| --- | --- | --- |
|  |  | |
| 15. | Feel that I need to do it enough | e.g. care more about the negative consequences of not trying to make lifestyle changes before my operation |
|  |  | |
| 16. | Believe that it would be a good thing to do | e.g. have a strong sense that I should make lifestyle changes before my operation |
|  |  | |
| 17. | Develop better plans for doing it | e.g. have clearer and well-developed plans for making lifestyle changes before my operation to make success more likely |
|  |  | |
| 18. | Develop a habit of doing it | e.g. getting into a pattern of making lifestyle changes before my operation without having to think too much about it |
|  |  | |
| 19. | Something else  (please specify): |  |

Thank you for taking the time to respond to this questionnaire.

## COM-B Self-Evaluation Questionnaire

When it comes to you personally providing remote support that targets lifestyle behaviour change to patients preparing for their operation, what do you think is needed for you to do it?

Please circle any of the items on the list that you think apply to you. You can circle as many or as few as you think appropriate. Some of the items may look strange, but that is just because we need to cover all areas – some which may not apply to you.

For each item you circle could you also say why you think it might be important for you in the free text box provided beneath.

I would have to....

Capability

| 1. | Know more about why it is important | e.g. have a better understanding of the benefits of supporting patients to make lifestyle changes to improve the quality of their sleep prior to surgery. |
| --- | --- | --- |
|  |  | |
| 2. | Know more about how to do it | e.g. have a better understanding of how to effectively support patients to make a number of lifestyle changes prior to surgery |
|  |  | |
| 3. | Have better physical skills | e.g. acquire/develop new skills to effectively support patients to make lifestyle behaviour changes prior to their surgery. |
|  |  | |
| 4. | Have better mental skills | e.g. learn how to reduce the likelihood that patients go off on tangents during discussions about lifestyle behaviour change prior to their surgery. |
|  | Ensuring regular updates including via email, telephone and occ face to face | |
| 5. | Overcome physical limitations | e.g. proceed to provide support to patients to make lifestyle behaviour changes prior to surgery when feeling tired. |
|  | Adapting information to suit different patient's, e.g upper body work at home in the chair using household items as opposed to attending a gym, continuing to use steps and stairs as part of daily exercise. E.g Reducing weight slowly and reducing cigarette consumption | |
| 6. | Overcome mental obstacles | e.g. overcome the urge to avoid providing support to a patient who has previously been resistant to changing their lifestyle behaviours |
|  |  | |
| 7. | Have more physical stamina | e.g. develop greater capacity to maintain physical effort, particularly following provision of support to a challenging patient. |
|  | This is where team work plays a greater part | |
| 8. | Have more mental stamina | e.g. increase mental capacity to discuss lifestyle behaviour change with patients, particularly following provision of support to a challenging patient. |
|  |  | |

I would have to....

Opportunity

| 9. | Have more time to do it | e.g. dedicate time to provide support to patients targeting lifestyle behaviour change. |
| --- | --- | --- |
|  | This needs to be part of any job plan | |
| 10. | Have the necessary materials | e.g. have materials available to me to help me target lifestyle behaviour change with my patients prior to surgery. |
|  | Mulitmedia choices help as well as addressing issues with a variety of scenarios and FAQ | |
| 11. | Have colleagues around me doing the same thing | e.g. be part of a group of colleagues who are also providing remote lifestyle behaviour change support to patients prior to surgery. |
|  | Having a sense of working in a team helps personal and team development | |
| 12. | Have triggers to prompt me | e.g. have reminders at strategic times to prompt me to use specific strategies to support lifestyle behaviour change in patients prior to surgery. |
|  |  | |
| 13. | Have support from others | e.g. have colleagues/supervisors supporting me to provide lifestyle behaviour change support to patients prior to surgery. |
|  | Peer group work with patient input is how I started our service | |

I would have to....

Motivation

| 14. | Feel that I want to do it enough | e.g. feel a sense of pleasure or satisfaction from providing support to patients to help them make lifestyle behaviour changes prior to surgery. |
| --- | --- | --- |
|  | There is no greater feeling than being thanked for supporting a patient's recovery | |
| 15. | Feel that I need to do it enough | e.g. care more about the negative consequences of not providing support to patients to make lifestyle behaviour changes prior to surgery. |
|  | The impact on patient’s lack of knowledge and skills to help themselves before surgery affects their recovery and nowhere is this more apparent than when they return to clinic post op. These appointments are always longer if the patients have not had adequate pre operative information | |
| 16. | Believe that it would be a good thing to do | e.g. have a strong sense that I should provide lifestyle behaviour change support to patients prior to surgery. |
|  | This is part of my ethos in working within orthopaedics, this is part of the holistic service we aim to provide | |
| 17. | Develop better plans for doing it | e.g. have clearer and well-developed plans for providing lifestyle behaviour change support to patients prior to surgery. |
|  | This is so important for consistency for each patient so that across all practitioners the same approach is used | |
| 18. | Develop a habit of doing it | e.g. getting into a pattern of providing lifestyle behaviour change support to patients prior to surgery without having to think too much about it. |
|  | This is already part of my role in supporting patients before during and after surgery | |
| 19. | Something else  (please specify): |  |

Thank you for taking the time to respond to this questionnaire.
