# Supplementary material for: Systematic development and feasibility testing of a multibehavioural digital prehabilitation intervention for patients approaching major surgery (iPREPWELL): A study protocol
Source: PLoS One. 2022 Dec 27;17(12):e0277143. doi: 10.1371/journal.pone.0277143 (PMC9794053; doi:10.1371/journal.pone.0277143)
Supplement: S2 File — (DOCX) [file pone.0277143.s002.docx]

Stage One, 1:1 Interviews Patients – Topic Guide

**Important** to consider, prior to interview, patient’s: [a] stage in surgical pathway (whether pre/post-operative) and [b] their involvement in receiving lifestyle advice preoperatively.

**Before recording/scene-setting:** We are designing a digital programme to help patients make lifestyle changes to better prepare for surgery. By ‘lifestyle’ we mean health and wellbeing advice and support to make improvements with nutrition, exercise, sleep, smoking, alcohol consumption and mental wellbeing. And by ‘digital programme’ we mean that the programme of support will be accessed through the internet using a desktop computer, mobile phone or tablet-device.

**Openers:**

We have contacted you for an interview because we are aware that you are [due to have/have had] an operation

1. When preparing for surgery, did/have you receive/[d] any lifestyle advice/information or specific support?
2. What kind of advice/information did you receive? Who provided it?
3. Did you find the advice/information useful? Was there anything else you needed to know? Was the information you were given understandable? Was it clear? How did it inform the way in which you prepared for your operation, if at all?

Capability

1. There are many benefits to making lifestyle changes before surgery. Do/did you feel you have/had a good understanding of why it is/was important to make lifestyle changes before surgery? Could you explain your answer?
2. Based on your understanding currently/at the time before surgery, do/did you feel you [are/were] able to make the necessary lifestyle changes to help you prepare for surgery?

Prompt: Or is/was there something else you would need/have needed? (What would that be/have been?)

1. If you were using a digital programme, like the one we are designing, what ‘skills’ do you think you would need to access and use the programme to help you make lifestyle changes before surgery?

Prompt: These can be:

- 1. Physical and ‘technical’ skills (e.g., IT literacy)
  2. Psychological or ‘soft’ skills (e.g., communication, asking for help)

1. Sometimes making lifestyle changes can be tiring and stressful.. [Looking back] Do you think you would find/have found making lifestyle changes more or less physically tiring and/or stressful, if you were using a digital programme?
2. While using a digital programme, what additional support or skills would you need/have needed to help you overcome these kinds of ‘physical limitations’ or challenges when making lifestyle changes ahead of surgery?
3. Similarly, feeling tired and stressed can sometimes make us feel like giving-up on making lifestyle changes. Do you think using a digital programme would have helped with feeling emotionally drained when making lifestyle changes, or more so? Could you explain your answer?

1. While using a digital programme, what additional support or skills would you need/have needed to help you overcome any emotional or mental obstacles or challenges when making lifestyle changes?

**Opportunity**

1. Do/Did you feel you have/had enough time to make lifestyle changes before your operation?

Prompt:

- Do/did you have enough time in the day? Enough time before surgery?
- If ‘no’: Is/Was it possible to make more time?
- How do you believe you could make/have made more time to make lifestyle changes before surgery?

1. As part of the digital programme, patients will need to use a computer, mobile phone or tablet device with access to the internet. Do you have access to devices like these? Would you need/have needed some help in accessing one? Would you need/have needed some help in using this device?
2. If you were using a digital programme, do you think you would find/have found it useful [at the time] to know of other patients who are/were also using the digital programme?

Prompt: And would you [like/have liked] the programme to connect you with other patients while using it?

1. When using the digital programme to make lifestyle changes, do you think it would be/have been helpful to receive reminders or prompts to make lifestyle changes? (e.g., an alert or prompt to help you remember)

Prompts: What kind of reminders do you think would be/have been most helpful? [e.g., text messages, emails, phone calls]

1. Outside of the digital programme, support to make lifestyle changes can also be important for some people. What support [if any] from other people would you find/have found helpful? [e.g., healthcare professionals, other patients, family, friends etc.,]

**Motivation**

1. In preparation/When you were preparing for surgery, do you feel that you want/did want to make lifestyle changes?

Prompt:

- Why was this important to you?
- And would a digital programme help you feel motivated to want to make lifestyle changes?

1. [At the time] Do/Did you feel that you need/needed to make lifestyle changes before surgery? Can you explain your answer?

Prompt:

- Can you think of/are you aware of any negative consequences of not making lifestyle changes before surgery? [e.g., social pressures]
- Would you feel you would need/have needed to use a programme like this in order to make those lifestyle changes?

1. [At the time] Do/Did you believe it would be a good thing to make lifestyle changes before your operation? If you were offered a digital programme by a healthcare professional – would this give/have given you a strong sense of: ‘making lifestyle changes is something you should be doing?’

Prompt: Can you tell me why making some lifestyle changes might not be a good idea for you personally? [e.g., social pressures]

1. In order to make lifestyle changes before your operation, do you think [having/having had] a well-developed plan would help you to reach your lifestyle goals? If so, could you talk me through what this plan would have to include to work for you? Would you feel/have felt motivated to use a digital programme to help develop this plan?
2. Sometimes getting in to a routine can help you achieve your lifestyle goals. Would you be/have been interested in using a digital programme to help you plan your lifestyle goals in a way that would help you develop a routine? Could you explain your answer?

Stage One, 1:1 Interviews HCPs – Topic Guide

**Important** to consider prior to interview that: the provision of remote support or promotion for prehabilitation uptake will be **relative to the role of HCP and therefore needs to be considered prior to interview.**

Before recording – scene-setting: **The intervention will require the perioperative team to offer and promote the programme to patients preoperatively, and this will include providing positive reinforcement to patients using it during consultations. A team of HCPs will be trained to oversee and support patients using the programme.**

**Openers:**

1. Can you tell me a bit about your role?
2. At what stages do you engage with patients in the preoperative pathway?

- Prompt: How much time do you usually have with each patient altogether?

1. Do you already provide lifestyle advice to patients? What does that entail?
2. What could you foresee your role being, if a programme like this were made available to patients in the preoperative pathway at your hospital?
3. Who in the healthcare team could or should have a role in promoting a programme like this to patients?
4. Who in the healthcare team could or should oversee patients using a programme like this?

**Capability**

1. When [remotely supporting patients to use/promoting] a digital prehabilitation programme, what additional knowledge would you need in order to understand the purpose of the intervention?
2. What specifically would you need to know about the digital prehabilitation programme to effectively [support patients to use it/promote it to patients]?
3. What additional skills and competencies would you need to effectively [remotely support patients to use/promote] a digital prehabilitation programme?

- Prompt: What physical and technical skills would you need? (e.g., IT literacy)
- Prompt: What psychological or ‘soft’ skills would you need? (e.g., communication, negotiation)

1. To what extent do you feel you need to develop skills to overcome your own physical limitations when [promoting/supporting] the use of a digital prehabilitation programme? (For example, when feeling physically tired, particularly if patients haven’t responded positively)

Prompts: work, time, environment, space, being face-to-face or over telephone – physical practicalities

1. Often, we are hesitant to offer an intervention to patients who have previously been resistant. To what extent do you feel you need training on how to overcome your own mental obstacles when [promoting/supporting] use of a digital prehabilitation programme to your patients?
2. [Promoting use of an intervention or supporting patients] to use an intervention can be physically tiring. How useful would you find training on how to maintain your physical effort when promoting or supporting use of a digital prehabilitation intervention?
3. Promoting use of an intervention or supporting patients to use an intervention can be mentally draining, particularly when patients do not engage. How useful would you find training on how to increase mental capacity to [promote the use of/support patients to use a] digital prehabilitation programme to patients prior to surgery?

**Opportunity**

**Within your working role:**

1. How much time realistically do you currently have to [promote use of/support patients to use a] digital intervention?

- Follow-up: How likely are you to dedicate more time to doing this, if it impacts positively on patient outcomes?

1. To what extent do you feel you have the necessary resources, materials or equipment to [promote use of/support patients to use a] digital prehabilitation intervention (prompt: if needed ask for more depth to the response)?
2. To help you [promote/ support patients to use] a digital prehabilitation programme, to what extent would you need colleagues around you doing the same thing?

- Prompt: Why/explain answer

1. To what extent do you feel you would need to be prompted to [promote the use of/support patients to use] a digital prehabilitation programme?
2. To what extent do you feel you require support from others to [promote use of/support patients to use a] digital prehabilitation intervention?

- Prompt: If needed ask for more depth to the response, for example what type of support are they looking for?

**Motivation**

**On a personal note, to what extent:**

1. Do you feel that you would want to [promote use of/support patients to use] a digital prehabilitation programme?
2. Do you feel that you would need to [promote use of/support patients to use] a digital prehabilitation programme?
3. Do you believe that it would be a good thing to [promote use of/support patients to use] a digital prehabilitation programme?
4. Do you believe that you would need to develop better plans to [promote use of/support patients to use] a digital prehabilitation programme?
5. Do you feel you would need to develop a habit to effectively [promote use of/support patients to use] a digital prehabilitation programme?
