# Supplementary material for: Systematic development and feasibility testing of a multibehavioural digital prehabilitation intervention for patients approaching major surgery (iPREPWELL): A study protocol
Source: PLoS One. 2022 Dec 27;17(12):e0277143. doi: 10.1371/journal.pone.0277143 (PMC9794053; doi:10.1371/journal.pone.0277143)
Supplement: S3 File — (DOCX) [file pone.0277143.s003.docx]

| **Physical activity and exercise capacity assessment methods** | |
| --- | --- |
| Stature (m) | Standard outpatient clinical measurement |
| Body mass (kg) | Standard outpatient clinical measurement |
| Body mass index (BMI) | Calculated as body mass (kg)/ stature squared (m^2^) |
| Resting heart rate | Standard outpatient clinical measurement using pulse oximeter |
| Resting blood pressure (mmHg) | Standard outpatient measurement using non-invasive blood pressure cuff |
| Resting oxygen saturation SPO2 (%) | Standard outpatient clinical measurement using pulse oximeter |
| Body composition | BodyStat 1500 bioimpedence device as per manufacturer instructions (BodyStat ltd, Douglas, UK) |
| **Exercise capacity assessments** | |
| 6-minute walk test (m) | Conducted using European Respiratory Society/ American Thoracic Society protocol [1] |
| 30 second sit-to-stand test (Repetitions) | Conducted using Jones et al [2] protocol |
| Grip strength (kg) | Conducted using Trampisch et al [3] protocol using Jamar Dynamometer (Patterson medical, Saint Paul, Minnesota, US) |
| Maximum inspiratory pressure (cmH2O) | Conducted using Silva et al [4] protocol using Powerbreathe K-series device (Powerbreathe, Southam, UK) |

1. Holland AE, Spruit MA, Troosters T, Puhan MA, Pepin V, Saey D, McCormack MC, Carlin BW, Sciurba FC, Pitta F, Wanger J. An official European Respiratory Society/American Thoracic Society technical standard: field walking tests in chronic respiratory disease. European Respiratory Journal. 2014; 44(6): 1428-46.
2. Jones CJ, Rikli RE, Beam WC. A 30-s chair-stand test as a measure of lower body strength in community-residing older adults. Research quarterly for exercise and sport. 1999 Jun 1;70(2):113-9.
3. Trampisch US, Franke J, Jedamzik N, Hinrichs T, Platen P. Optimal Jamar dynamometer handle position to assess maximal isometric hand grip strength in epidemiological studies. J Hand Surg Am. 2012 Nov;37(11):2368-73. doi: 10.1016/j.jhsa.2012.08.014. PMID: 23101534.
4. Silva PE, de Carvalho KL, Frazão M, Maldaner V, Daniel CR, Gomes-Neto M. Assessment of maximum dynamic inspiratory pressure. Respiratory Care. 2018 Oct 1;63(10):1231-8.
