# Supplementary material for: Systematic development and feasibility testing of a multibehavioural digital prehabilitation intervention for patients approaching major surgery (iPREPWELL): A study protocol
Source: PLoS One. 2022 Dec 27;17(12):e0277143. doi: 10.1371/journal.pone.0277143 (PMC9794053; doi:10.1371/journal.pone.0277143)
Supplement: S4 File — (DOCX) [file pone.0277143.s004.docx]

Study data will be securely collected, stored, and analysed in accordance with UK data protection laws (data protection act 2018 and general data protection regulations 2018). Informed consent will be obtained from participants for all aspects of data handling. No individual participants will be identifiable in publications or presentations of study data.

**Stage 1 Data handling**

Participant paper CRFs, completed COM-B questionnaires, interview transcripts, and workshop facilitator notes will be stored securely in site files in locked offices. Paper data will be converted into a password protected electronic format (e.g., Microsoft Excel/Microsoft Word).

Interview and workshop audio recordings will be transferred from encrypted recorders and stored securely on NHS servers with password protection. Audio files will be converted to a pseudo anonymised password protected transcript file by an approved transcription service for subsequent transfer and analysis. Once transcribed, original audio files will be deleted.

Direct access to pseudo anonymised data will be granted to authorised study team members, representatives from the study sponsor, academic institutions, and the regulatory authorities to permit study-related monitoring, audit, and inspections.

**Stage 2 data handling**

The planned digital intervention has undergone a full data protection impact assessment (DPIA) by the sponsor.

The partner web developer (Hark 2 limited) is registered with the UK information commissioner (ref: ZA435653) and hold current cyber essential certification. Hark 2 have an established track record creating similar web-based interventions for clinical use in the UK National Health Service (NHS). Hark 2 will not act as a data processor and will be unable to access participant data in a non-encrypted format.

The digital intervention will be held on a secure server with multiple security measures in place including: Following of good security practices during iterative development (e.g., OWASP top 10); all participant data encrypted at rest with AES 256 CBC cypher; all participant data encrypted during transmission via TLS; strong password policy enforced for all patient participant, HCP participant and research team member/administrator accounts; auto logout when users are idle; multi-factor authentication for admin accounts; auto locking of administrator accounts after a set period of inactivity (e.g. three months without logging in); and forced password changes at set intervals.

Participants will be asked to provide consent to utilise a commercially integrated wearable device and accompanying smartphone application in accordance with manufacturer instructions and data protection policies. Biometric data e.g., heart rate and step count will be collected and stored by the manufacturer in accordance with those policies. The digital intervention platform will obtain data from the device manufacturer via a 3^rd^ party application programming interface (API). These data will be securely stored alongside other programme data.

Data held by the platform will be downloaded to secure site servers for analysis or inspection with routine access by study team members only. Direct access will be granted to authorised representatives from the Sponsor, academic institutions, and the regulatory authorities to permit study-related monitoring, audit, and inspections.

Paper HCP participant diaries and semi-structured interview data will be handled in keeping with procedures reported for stage 1 of the study.
